# First Complete Genome Sequence of *Bean common mosaic necrosis virus* from East Timor

**DOI:** 10.1128/genomeA.01049-16

**Published:** 2016-09-29

**Authors:** Solomon Maina, Owain R. Edwards, Luis de Almeida, Abel Ximenes, Roger A. C. Jones

**Affiliations:** a School of Plant Biology, Faculty of Science, University of Western Australia, Crawley, Western Australia, Australia; b Institute of Agriculture, Faculty of Science, University of Western Australia, Crawley, Western Australia, Australia; c Cooperative Research Centre for Plant Biosecurity, Canberra, Australian Capital Territory, Australia; d CSIRO Land and Water, Floreat Park, Western Australia, Australia; e Seeds of Life Project, Ministry of Agriculture and Fisheries, Dili, East Timor; f DNQB-Plant Quarantine International Airport Nicolau Lobato Comoro, Dili, East Timor; g Department of Agriculture and Food Western Australia, South Perth, Western Australia, Australia

## Abstract

We present here the first complete *Bean common mosaic necrosis virus* (BCMNV) genomic sequence isolated from virus-infected common bean (*Phaseolus vulgaris*) in East Timor, and compare it with six complete BMCNV genomes from the Netherlands, and one each from the United States, Tanzania, and an unspecified country. It most resembled the Netherlands strain NL-8 genome.

## GENOME ANNOUNCEMENT

To examine possible connections between viruses infecting crops in northern Australia and Southeast Asia, common bean (*Phaseolus vulgaris*) plants with virus-like symptoms were sampled in East Timor and northwest Australia (1–5). *Bean common mosaic necrosis virus* (BCMNV; genus *Potyvirus*, family *Potyviridae*) is spread nonpersistently by several aphid species and transmitted through infected seeds (6). It causes mosaic and curling of leaves and plant dwarfing and is important in common bean production in North America, East Africa, and Europe (6, 7). Before 1992, BCMNV was included within *Bean common mosaic virus* (8). It has not been reported from East Timor, northwest Australia (9), or the rest of Australia (6). Nine complete BCMNV genomes are available in GenBank: six from the Netherlands (including one duplicated genome), and one each from Tanzania, the United States, and an unspecified country (6, 7, 10). BCMNV was detected in only one sample (TM70) collected in May 2015 from the Aileu district of East Timor. It was sequenced, and a complete genome was obtained.

Fifteen East Timorese samples were blotted onto Fast Technology for Analysis of nucleic acids (FTA) cards (11). Total RNA was extracted from these cards using ZR Plant RNA MiniPrep kit (Zymo Research). The total RNA extracts were treated with RNase-free DNase (Invitrogen) and measured using Qubit (Invitrogen). RNA integrity was confirmed using RNA ScreenTape (TapeStation 2200, Agilent Technologies). Libraries were prepared from total RNA using a TruSeq stranded Total RNA sample preparation Ribo-Zero plant kit (catalogue no. RS-122-2401, Illumina). The final size and concentration of each library was verified using Qubit and D1000 ScreenTape (TapeStation 2200). Sequencing was by Macrogen Inc. using HiSeq 2500 with a TruSeq SBS kit version 4 (Illumina) with 151 cycles of paired-end reads. Reads were then assembled and genomes annotated using CLC Genomics Workbench version 6.5 (CLC bio) and Geneious version 8.1.7 (Biomatters) (12). Further alignment was by MAFFT (13).

FTA card sample TM70 yielded 2,248,678 reads and, after trimming, 1,948,678 remained. *De novo* assembly generated 108 contigs, and 858,904 reads were mapped to the contig of interest with coverage of 12,871×. The final complete genome sequence length was 9,640 nucleotides (nt) containing the 5′ (157 nt) and 3′ (242 nt) untranslated regions. The new sequence coded for 10 proteins, as with other potyviruses (14). A BLAST-based search using a pairwise sequence comparison tool (15) revealed that TM70 most resembled the Netherlands strain NL8 genome, accession number HQ229994 (7). Pairwise nucleotide alignment of these two isolates was 98.0% and was well within the 76% species demarcation limit for *Potyvirus* genomes (16, 17). Since no BCMNV was detected in any Australian samples, further sampling is needed to establish whether BCMNV has spread to Australia from nearby Southeast Asian countries. Comparison of any Australian genomic sequences found with ones from neighboring countries would be required.

### Accession number(s).

The sequence was deposited in GenBank under the accession number KX302007.
